# Hyaluronic Acid: The Influence of Molecular Weight on Structural, Physical, Physico-Chemical, and Degradable Properties of Biopolymer

**DOI:** 10.3390/polym12081800

**Published:** 2020-08-11

**Authors:** Petr Snetkov, Kseniia Zakharova, Svetlana Morozkina, Roman Olekhnovich, Mayya Uspenskaya

**Affiliations:** Institute BioEngineering, ITMO University, Kronverkskiy Prospekt, 49A, 197101 St. Petersburg, Russia; zakharova_kseniia@lenta.ru (K.Z.); Morozkina.Svetlana@gmail.com (S.M.); r.o.olekhnovich@mail.ru (R.O.); mv_uspenskaya@mail.ru (M.U.)

**Keywords:** biocompatibility, degradability, hyaluronic acid, molecular weight, structure, receptors, viscosity, water polymer solution

## Abstract

Hyaluronic acid, as a natural linear polysaccharide, has attracted researchers’ attention from its initial detection and isolation from tissues in 1934 until the present day. Due to biocompatibility and a high biodegradation of hyaluronic acid, it finds wide application in bioengineering and biomedicine: from biorevitalizing skin cosmetics and endoprostheses of joint fluid to polymeric scaffolds and wound dressings. However, the main properties of aqueous polysaccharide solutions with different molecular weights are different. Moreover, the therapeutic effect of hyaluronic acid-based preparations directly depends on the molecular weight of the biopolymer. The present review collects the information about relations between the molecular weight of hyaluronic acid and its original properties. Particular emphasis is placed on the structural, physical and physico-chemical properties of hyaluronic acid in water solutions, as well as their degradability.

## 1. Introduction

Hyaluronic acid (HA), as a member of hyaluronan family, was first discovered by K. Meyer and John W. Palmer in 1934 [1], and nowadays continues to attract careful attention on the part of chemists, biochemists, bioengineers, and other investigators from various scientific areas. HA is an essential component of the extracellular and pericellular matrixes, and can also be found inside cells [2]. The occurrence of hyaluronic acid in tissues varies: for instance, rooster’s combs contain 7.50 mg/mL, human navel cords (gelatin of Wharton)—4.10 mg/mL, human joint synovial fluid—1.50–3.60 mg/mL, vitreous humor—0.14–0.34 mg/g, human dermis and epidermis—0.20–0.50 and 0.10 mg/g, respectively [3]. The turnover of hyaluronic acid in vertebrate tissues on average is equal to 5 g per day, and is provided by biosynthesis and enzymatic degradation [4]. Meanwhile, the turnover of hyaluronic acid in the blood-flow reaches 30–100 mg per day [5].

Apart from the animal of origin, hyaluronic acid can be separated based on bacteria, for example, from *Streptococcus* genus (*uberis, equisimilis, zooepidermicus, pyogenes, equi*), *Pasteurella multocida* [6,7,8,9,10], and *Corynebacterium glutamicum* [11]; from the green algae Chlorella purposely infected by the Chlorovirus [6,7,12]; Saccharomycetes (*Cryptococcus neoformans* [6,7]); and from molluscan shellfish, such as the bivalve mollusc *Mytilus galloprovincialis* [6,13]. At the same time, hyaluronic acid has not been disclosed in fungus, insects, or plants [6,14].

Note that hyaluronic acid is usually obtained from bovine vitreous humors, rooster combs, the skin of sharks, and human umbilical cords [15]. However, animal origin hyaluronic acid contains endotoxin and protein residuals, which possess immunogenic effects [14,15]. Thus, 1 mg of hyaluronic acid from human navel cord and from bovine vitreous humor could include >100.0 EU endotoxin and approximately 47.7 and 36.2 μg protein, respectively. By contrast, hyaluronic acid from rooster comb contains 23.0 EU endotoxin and 1.0 μg protein per 1 mg of polymer [16]. At the same time, bacterial technology makes it possible to obtain high-purity hyaluronic acid with low protein and endotoxin levels [15]. Therefore, bacterially derived hyaluronic acid purchased from Sigma and Genzyme includes only 1.0–1.6 μg protein and approximately 0.02 EU endotoxin per 1 mg hyaluronic acid [16]. Nevertheless, the level of immunogenic effect of protein residuals in bacterial hyaluronic acid could be greater than in animal hyaluronic acid despite the low summary protein content [17].

It is obvious that molecular weight of hyaluronic acid depends on the source. Consequently, hyaluronic acid from animal materials has a very high molecular weight (up to 20,000 kDa). For example, rooster combs contain hyaluronic acid with 1200 kDa, the navel cords—3400 kDa, bovine vitreous humors—770–1700 kDa. By contrast, bacterial hyaluronic acid has a molecular weight between 1000 and 4000 kDa; however, the enzymatic technique makes it possible to obtain polysaccharides with a range of molecular weight between 550 kDa and 2500 kDa [18]. The molecular weight of hyaluronic acid also depends of some other conditions: for instance, in human normal synovial fluid, it is equal to 6000–7000 kDa, while in rheumatoid fluid, the molecular weight is less, and is equal to 3000–5000 kDa [19,20].

The biological effects of hyaluronic acid depend heavily on molecular weight. Hyaluronic acid with molecular weights from 0.4 to 4.0 kDa acts as an inducer of heat shock proteins, and has a non-apoptotic property. Polysaccharides with a molecular weight equal to 6–20 kDa possess immunostimulatory, angiogenic, and phlogotic activities. Hyaluronic acid with a molecular weight of 20–200 kDa takes part in biological processes such as embryonic development, wound healing and ovulation. By contrast, high molecular weight hyaluronic acid (>500 kDa) has anti-angiogenic activity, and can function as a space filler and a natural immunologic depressant [21].

The fact that the molecular weight of hyaluronic acid may vary its biological properties is the current subject of interest. The drastic difference in its functions is the reason that for medical applications, preference is given to low-polydispersity or monodisperse HA. Preparation of monodisperse hyaluronic acid is achieved by successive cycles of degradation and subsequent assembly of HA chains [22]. Additionally, the mechanisms of interaction of hyaluronic acid of various molecular weights with receptors on the cell surface are currently being actively studied. It has previously been suggested that HA of different MW may affect the same receptors differently; however, recent study refutes this theory [23]. On the other hand, it has been shown that HA of very high molecular weight (6000 kDa) produced by naked mole-rat suppresses the signaling of CD44, which results in altered expression of a subset of p53 target genes, thereby suggesting that HMWHA has the properties of a cytoprotective molecule [24], but there are differences in the genes regulated by p53 between different species so this investigation is restricted to human cells.

Evidently, the structural, physical, physicochemical and degradable properties of hyaluronic acid also depend on its molecular weight. For example, the increase in the molecular weight and concentration of hyaluronic acid in polymer solutions leads to the reinforcement of the three-dimensional network of the polymer. Consequently, it results in an increase in the solution viscosity and viscoelasticity [6]. In some cases, for example, in the electrospinning process, molecular weight, concentration, and viscosity are the key parameters providing the nanofibers obtaining [25].

There are a lot of studies dedicated to the properties of hyaluronic acid. Unfortunately, the vast majority of such papers touch upon one of several groups of polymer characteristics. Particular interest is aroused by the biological properties of materials based on hyaluronic acid. Still, for the development and technology of advanced wound healing [26], drug delivery systems with controlled release [27], and polymer scaffolds [28], knowledge of the molecular weight dependency on the abovementioned properties is necessary. Our paper is one of the first to collect brief data on the structure, viscosity, density, surface tension, cohesive/adhesive, hydrodynamic and degradable properties of hyaluronic acid.

## 2. Aqueous Hyaluronic Acid Solutions and Their Properties

### 2.1. Structural Properties

Hyaluronic acid is a linear heteropolysaccharide (glucosaminoglycan, mucopolysaccharide) with high molecular weight formed by regularly repeating residues of N-acetyl-D-glucosamine and D-glucuronic acid [1,29]. In a hyaluronic acid molecule, the D-glucuronic acid is associated with amino-sugar by β-(1 → 3)-glycosidic linking, and amino-sugar is connected with the D-glucuronic acid by a β-(1 → 4)-glycoside tieup [17]. Obviously, that structure of the monomeric unit (named as the primary structure) is not dependent on the molecular weight of hyaluronic acid.

The existence of polar and apolar segments in the polymer structure affords hyaluronic acid the capability to chemically interact with various chemical agents [17], for instance, with metachromatic dyes, which find application in clinical examinations [30], and chitosan, which makes it possible to obtain a new class of materials based on polyelectrolyte complexes [31,32].

**Figure 1:**
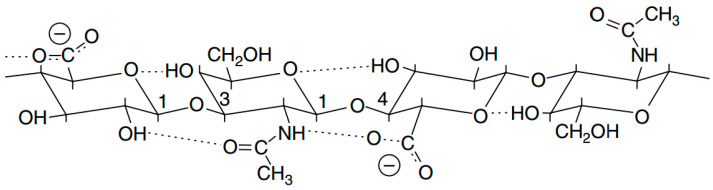
The schematic representation of hydrogen bond formation [33]. Copyright © (1999) National Academy of Sciences. Reproduced from [17], with permission from John Wiley and Sons, 2020.

Hyaluronic acid forms hydrogen bonds, which, on the one hand, could poise the macromolecule in solutions, but, on the other hand, give rise to rigidity in the polymer system, which, finally, specify the properties of hyaluronic acid solutions. Figure 1 demonstrates the potential hydrogen bonds that could form both within one macromolecule, and between the adjoined molecules. Note that an aqueous molecule could be a bridge between the two connected functional groups [17,33]. Eventually, such primary structure and hydrogen bonds help to form secondary and tertiary structures [33,34].

Hyaluronic acid and its salt, with ammonium ions, magnesium, and alkaline metals, have good solubility in water and possess a high level of viscosity even at low polymer concentrations [17]. Moreover, hyaluronic acid in solution could organize a three-dimensional cellular structure with enormous dimensions at concentrations of less than 1 μg/mL [34]. By contrast, biopolymers can organize pseudo-gels when concentrations are equal to or above 1.0 wt.% [17,19,20]. However, hyaluronic acid with high molecular weight equal to 5.0 MDa at concentrations greater than 0.1 mg/mL forms entangled polymer networks, but hyaluronic acid solutions do not have prolonged mechanical integrity [12]. Salts of hyaluronic acid with cations possessing two and more valence numbers have substantial insolubility in water. Additionally, if such ions are introduced into hyaluronic acid solutions, intermolecular cross-links are constituted, resulting in the development of a gel with great water content [17].

**Figure 2:**
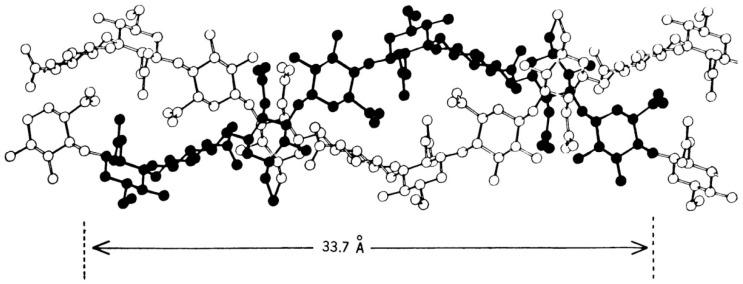
Schematic representation of the antiparallel helix structure of hyaluronic acid. Reproduced from [36], with permission from The American Association for the Advancement of Science, 2020.

It is known that the macromolecule of hyaluronic acid in solution could organize the left-oriented individual or twin spiral [34]. Study confirms that hyaluronic acid K and Na salts demonstrate a twin helix structure in solution [35]; moreover, that helix has antiparallel left-oriented strands [17,36]. Figure 2 demonstrates the antiparallel double structure of hyaluronic acid.

It is interesting to note that apart from the double helix structure, there is a supra-helical organization of hyaluronic acid that could also be detected [37]. As mentioned above, with an increase in hyaluronic acid concentration, an intramolecular polymer network could be formed. In the dilute hyaluronic acid solution, the biopolymer could be a separate molecule, but with the increase of polymer concentration, three-dimensional web structures are organized. This is due to the incapacity of the adjacent hyaluronic acid molecules to have several varying and separate forms. Note that free hyaluronic acid molecules could be folded into the roll [17].

The secondary hyaluronic acid structure bears a likeness to flat bands transformed into a helix or twisted into a sheet [33,34] (see Figure 2). It is confirmed that intermolecular hydrogen bonds affect the structure of hyaluronic acid [17,29,34,35,36]. In the diluted hyaluronic acid solutions, the macromolecules have semi-rigid coiled chains and could form helix bands and even helical rings. Due to forming a rigid helix, the macromolecules of hyaluronic acid attract a great quantity of water and organize the wide domains of the tertiary polymer structure [17,35,36,38,39].

For a semi-flexible hyaluronic acid, the amount of the expansion that each polymer chain fills is enormous. The water molecules that are unbonded with the polymer constitute the majority of the volume occupied by hyaluronic acid. The polymer configuration is constantly in a state of motion and change, but the water increases the effective size of each hyaluronic acid macromolecule in view of the fact that the frictional engagement with closely pitched chains of biopolymer influence the solvent movement. Moreover, the effective spheroidal volume of the hyaluronic acid segment increases with an increase in the molecular weight of the polymer (to the power of 1.8). In other words, if the length of the polymer increases, the average density decreases due to the increase in mass being slower than that of the volume. Thus, the chains of hyaluronic acid with high molecular weight (more than 1000 kDa) occupy an extremely large volume [40].

Note that the hyaluronic acid hydrodynamic volume is generally analyzed at an ionic force that is equal to the physiological level. In this case, the charges of the carboxylate groups in the hyaluronic acid segment are almost wholly enclosed from each other, so the repulsive interaction between them could not noticeably broaden the volume of the polymer coil. If a polymer solution has a sodium chloride content less than about 0.15 M, the electrostatic repulsion expands the hydrodynamic volume of each hyaluronic acid macromolecule and increments the repulsion between them.

The hydrodynamic size of the hyaluronic acid depends on the molecular weight (see Table 1). The hydrodynamic size as the final result effects the polymer concentration, as polymer coils start to interpenetrate each other.

Note that the structure of hyaluronic acid in solution is very sensitive to pH; the addition of acids or alkalis leads to the displacement of the balance between repelling and attractive forces of polymer chains [6,41]. When the pH is more than 11.0 and less than 4.0, hyaluronic acid depolymerizes [6,42]. However, in alkaline solutions, the degradation effect is strongly pronounced because of the breakdown of hydrogen bonds, which play an important role in the structural organization of hyaluronic acid [41,43,44].

### 2.2. Rheological Properties

Obviously, rheological properties of hyaluronic acid solutions depend on the structure of the biopolymer and its polyelectrolyte character [33,45,46,47]. Hyaluronic acid solutions could be characterized as non-Newtonian liquids with shear-thinning and elastoviscous behavior [6,48]. The shear-thinning profile of hyaluronic acid solutions is attributable to several reasons: to the destruction of the intramolecular hydrogen bonds, and to hydrophobic effect with the shear rate increases. The last case is caused by deformation of hyaluronic acid molecular chains and their implication in the flow direction, resulting in reduction of the solution viscosity [41,47]. Interestingly, hyaluronic acid solutions do not demonstrate thixotropic properties; if the shear rate becomes smaller and finally is next to nothing, hyaluronic acid molecular chains assume the initial structure. Moreover, the viscosity curve goes through the same dots in the reverse direction [45]. Consequently, the destruction of the three-dimensional polymer network is reversible and could be considered a transient phenomenon. The viscosity of hyaluronic acid solutions is unique and retains its relevance and significance in physiological and biochemical processes, as well as during development of therapeutic, medical, cellular, bioengineering, cosmetic, and food application of hyaluronic acid.

**Figure 3:**
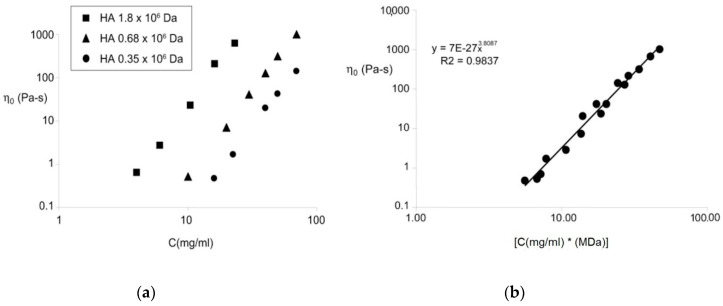
The dependency of the zero shear viscosity of hyaluronic acid on concentration for various molecular weights (**a**); on the [biopolymer concentration × molecular weight] (**b**). Reproduced from [49], with permission from John Wiley and Sons, 2020.

Falcone, S.J. et al. [49] used three types of hyaluronic acid with various molecular weights: low (350 kDa), medium (680 kDa), and high (1800 kDa). The dependence of the zero shear viscosity η0 (Pa∙s) on the hyaluronic acid concentration is demonstrated in Figure 3a. Figure 3b shows that the linear plot describing the dependency of the zero shear viscosity on concentration is multiplied by the molecular weight of hyaluronic acid. The experimental data show an evident relationship between the viscosity and both molecular weight and concentration. The results correlate with the previous full-scale study [50]. Interestingly, that two-fold increase/decrease of hyaluronic acid concentration or molecular weight leads to a ten-fold respective change of the zero shear viscosity of polymer solution [50] (see Table 2).

**Figure 4:**
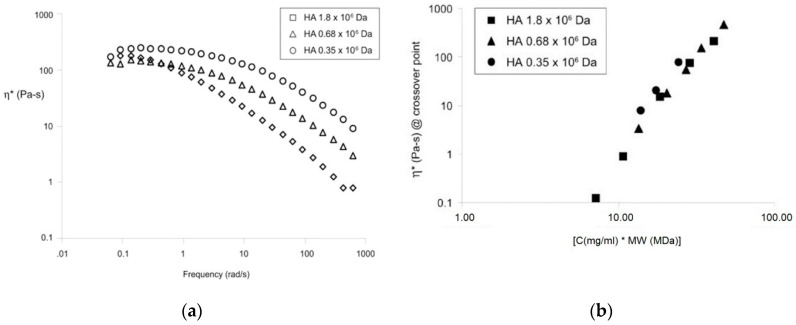
The dependency of the complex viscosity of frequency for hyaluronic acid with various molecular weights (**a**). The dependency of the complex viscosity at the crossover point on the [hyaluronic acid concentration × molecular weight] (**b**). Reproduced from [49], with permission from John Wiley and Sons, 2020.

Unfortunately, the zero shear viscosity of hyaluronic acid does not completely describe the unique properties of hyaluronic acid. Oscillation analysis makes it possible to obtain the dependency of the complex viscosity η* (as the sum of the storage modulus G′ and the loss modulus G″) and the frequency (see Figure 4a). The complex viscosity of solutions decreases with the increase in the molecular weight of hyaluronic acid. Additionally, the frequency and the modulus values of the crossover point of G′ and G″ increase with the decrease in the molecular weight of hyaluronic acid. Hyaluronic acid with high molecular weight possesses a longer relaxation time (passage from a mainly viscous behavior to an elastic behavior). With decrease in the molecular weight of hyaluronic acid, it takes less time for a three-dimensional polymer network to untwine and demonstrate principally viscous behavior [49]. The behavior difference between lower and higher molecular weight hyaluronic acid could also be explained by variation in the entwinement of the polymer chains [51].

Figure 4b demonstrates the function between the complex viscosities at the crossover point on the [hyaluronic acid concentration × molecular weight]. The authors note that hyaluronic acid solutions containing 10.0 mg/mL (680 kDa), 16.0 mg/mL and 22.5 mg/mL (350 kDa) did not show the cross-over points. Nonetheless, the complex viscosity at the cross-over point has a linear dependency on [hyaluronic acid concentration × molecular weight] [49].

**Figure 5:**
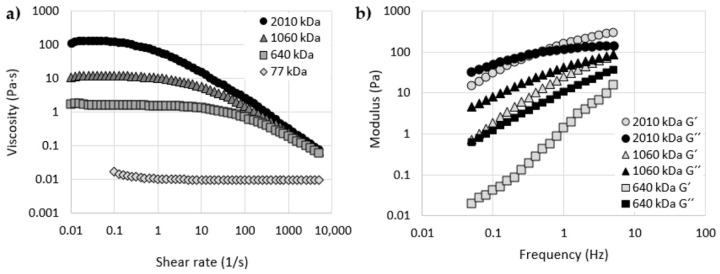
(**a**) The dependency of viscosity on shear rate for hyaluronic acid solutions with different molecular weights; (**b**) the dependency of storage modulus (G′) and loss modulus (G″) on frequency for hyaluronic acid solutions with various molecular weights. Reproduced from [52], with permission from MDPI, 2020.

Rebenda et al. [52] analyzed the rheological properties of four hyaluronic acid solutions with concentration of 20 mg/mL and with various molecular weights: 77 kDa, 640 kDa, 1060 kDa, 2010 kDa. The obtained results are demonstrated in Figure 5 and Table 3.

As mentioned earlier, hyaluronic acid solutions exhibit non-Newtonian shear thinning behavior. Figure 5a demonstrates that the solution with 2010 kDa hyaluronic acid has the highest viscosity reduction with increasing shear rate. By contrast, the shear thinning behavior of polymer solution with 77 kDa hyaluronic acid is feebly marked. 

Results of the frequency sweep analysis are shown in Figure 5b. Hyaluronic acid solutions with 640 kDa and 1060 kDa have a viscous-like character across the entire range of frequencies. On the other hand, the sample with 2010 kDa hyaluronic acid demonstrates a viscoelastic behavior with a crossover point equal to 0.4 Hz. Crossover frequency characterizes a transition point between viscous and elastic behavior. This parameter is important, especially for artificial synovial fluids, because such elastic matter is able to successfully absorb mechanical energy and preserve cartilage from damage or fretting.

Note that the higher the molecular weight of the hyaluronic acid, the higher the values of G′ and G″ (see Table 3). Moreover, the eventual crossover point is displaced to a lower level of frequency.

**Figure 6:**
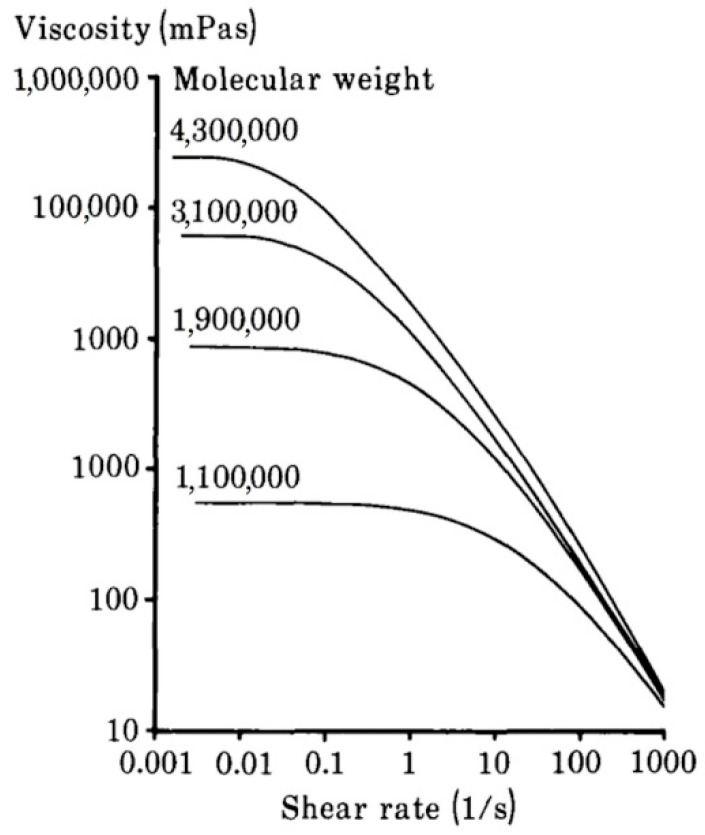
The effect of the molecular weight of hyaluronic acid on dynamic viscosity depending on the shear rate for the polymer solutions at a concentration equal to 10 mg/mL Adapted from [50], with permission from Taylor & Francis, 2020.

The dependency of dynamic viscosity on shear rate for hyaluronic acid solutions with various molecular weights is demonstrated in Figure 6. The polymer concentration is equal to 10 mg/mL in each case. It is worth noting that at high shear rates, the dynamic viscosity does not depend on the molecular weight of hyaluronic acid. The polymer macromolecules line up in the flow route, and the viscosity could be determined by polymer concentration [50]. This property makes it possible to extrude hyaluronic acid solutions through a narrow needle, for instance, for viscosupplementation [2,53].

**Figure 7:**
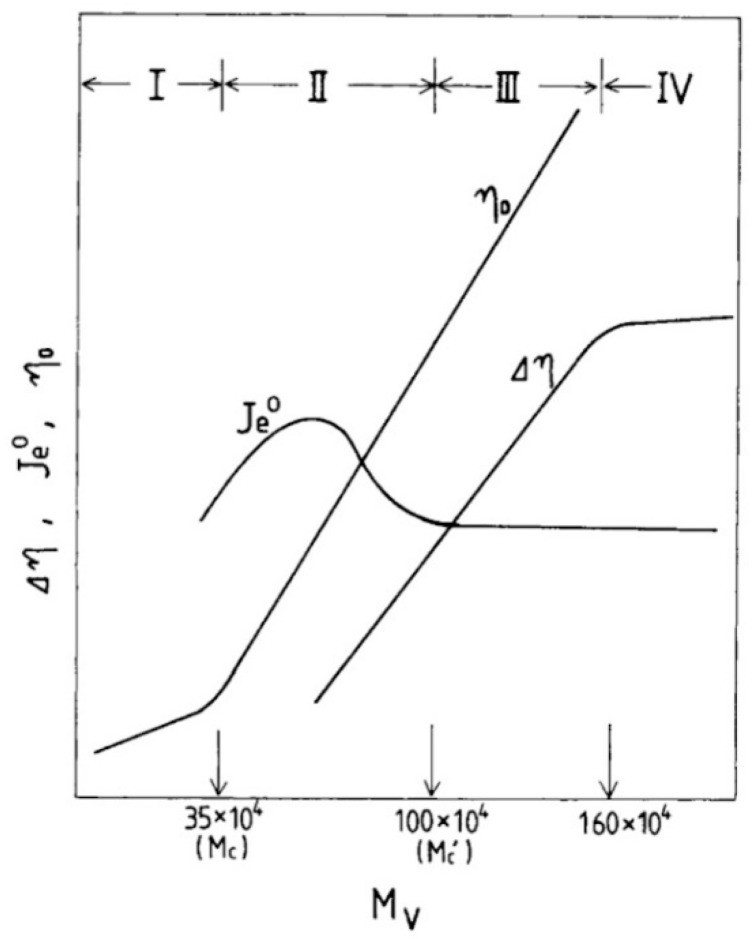
The schematic representation of four molecular weight sections into which the flow mechanism could be categorized based on the network structure. Here η_0_ is the zero shear viscosity; J_e_^0^—the steady shear compliance; and ∆η—the difference between η_0_ and the specific viscosity at the steady state. Reproduced from [54], with permission from John Wiley and Sons, 2020.

As mentioned above, the rheological properties of hyaluronic acid solutions are directly dependent on the biopolymer network structure. To summarize this dependency, Yanaki, T. and Yamaguchi, T. divided the molecular flow mechanism of 1.0% hyaluronic acid solutions into four regions (see Figure 7). In the first region, where the molecular weight of hyaluronic acid is less than 350 kDa, biopolymer molecules are dispersed in solution. In the second area (where the molecular weight is from 350 kDa to 1000 kDa), the occurrence of the weak entwinement of polymer chains is detected only by the zero shear viscosity η_0_, and the steady shear compliance J_e_^0^. The third region (where the molecular weight is from 1000 kDa to 1600 kDa) shows the presence of a strengthened polymer network, which is detectable by the constant J_e_^0^ and also the increase of η_0_. Moreover, the stress overshoot phenomenon is visible. The last region, where the molecular weight of hyaluronic acid is more than 1600 kDa, the three-dimensional polymer network is completed [54]. 

### 2.3. Surface Tension

Surface tension is not a common parameter of aqueous hyaluronic acid solutions, but it is a characteristic that could be useful in some cases. Some human diseases are accompanied by a decrease in the molecular weight of hyaluronic acid in the tissues involved. For instance, in primary open-angle glaucoma, the amount of HA decreases in trabecular meshwork, and an alteration in surface tension might be one of the consequences of the action of hyaluronic acid on the aqueous outflow [55,56,57,58]. The knowledge of changes in surface tension could help to better understand some degradation processes in the human body. 

A detailed scientific study dealing with the surface tension of hyaluronic acid aqueous solution in which biopolymer had different molecular weight was performed in 1995 [59]. The authors used three types of sodium hyaluronate (Na-HA): from bovine vitreous humor (*M*_W_ = 100 kDa), from human umbilical cords (*M*_W_ = 500 kDa), and from rooster combs (*M*_W_ = 4000 kDa). The main aim of this study was to compare the surface-active properties of Na-HA in deionized water, in Ringer’s lactate with/without bovine serum albumin, and in mock aqueous solutions. For the measurement of surface tension, the drop volume method was used. For samples of sodium hyaluronate with molecular weight equal to 100 kDa and 500 kDa, purification was achieved. This procedure could cause a decrease in the molecular weight of the biopolymer, and the authors mentioned the possibility of the further determination of molecular weight using the acid-urea polyacrylamide gel electrophoresis, but, unfortunately, there are no confirmatory data.

**Figure 8:**
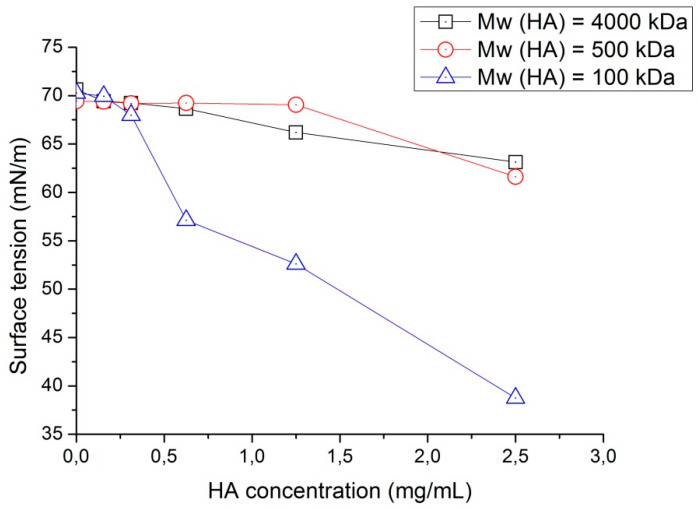
Surface tension of hyaluronic acid as a function of concentration in deionized water (plotted based on the experimental data from [59]).

Based on the above-mentioned study, the surface tension of sodium hyaluronate in deionized water greatly depends on concentration and molecular weight (Figure 8). Surface tension decreases with the increase of biopolymer concentration from 0.156 to 2.500 mg/mL. Na-HA with *M*_W_ = 100 kDa shows a noticeable decrease in surface tension with an increase in biopolymer concentration. By contrast, water solutions with sodium hyaluronate (*M*_W_ = 500 kDa and 4000 kDa) do not exhibit significant changes in surface tension with concentrations ranging from 0.156 to 1.250 mg/mL, but have a small recession at concentrations equal to 2.500 mg/mL (for second one) and equal to 1.250 (for the first one).

Unfortunately, the authors did not explain the reason for the significant decrease in surface tension with the increase of Na-HA with molecular weight equal to 100 kDa. It could be caused by the fact that solutions containing sodium hyaluronate with viscosity-average molecular weight up to 350 kDa are characterized by molecular dispersity. By contrast, biopolymer solutions with Na-HA possessing similar molecular weights with values no less than 1600 kDa demonstrate a saturated and complete network structure [54].

The next detailed study [60] demonstrated the effect of concentration and temperature on the surface tension of sodium hyaluronate water solution with sodium chloride at physiological concentrations. The authors used bacterial sodium hyaluronate with a molecular weight equal to 1630 kDa to obtain phosphate buffered solutions at concentrations from 0.5 to 3.5 mg/mL. In each solution, NaCl was added to normalize the osmolarity to the artificial tears value (~300 mOsm). Surface tension was measured using the pendant drop method.

**Figure 9:**
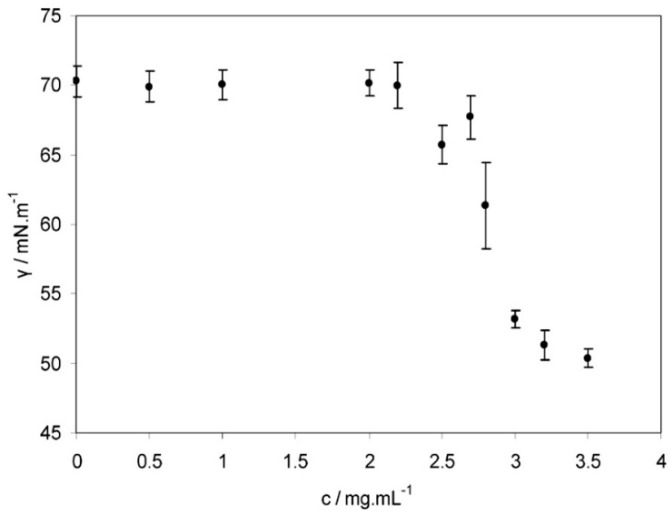
Surface tension of sodium hyaluronate solutions as a function of polymer concentration. Reproduced from [60], with permission from American Chemical Society, 2020.

Figure 9 demonstrates the equilibrium surface tension of sodium hyaluronate solutions as a function of concentration at 25 °C. 

The authors described three stages for the concentration dependence of surface tension, which correspond to structural changes described in the previous study [61]:

- Up to 2.2 mg/mL, the surface tension is equal to that of the phosphate buffered solution;
- from 2.2 mg/mL to 3.0 mg/mL, the surface tension undergoes a significant decrement;
- above 3.0 mg/mL, the surface tension exhibits a minor decrease.

The authors also emphasized the erratic behavior of the solutions with concentrations close to 2.4 mg/mL, which can be explained by the beginning of the entanglement process.

**Figure 10:**
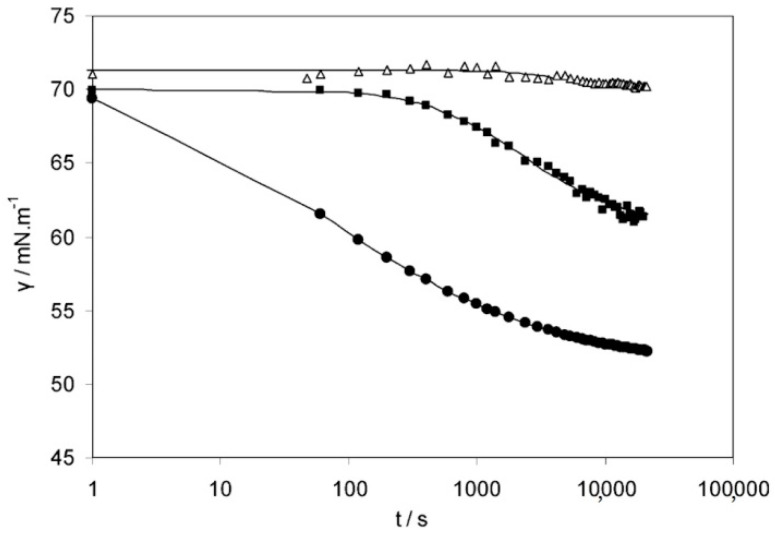
Dynamic surface tension of sodium hyaluronate solutions as a function of time: 2.0 mg/mL (∆), 2.8 mg/mL (■), 3.5 mg/mL (●). Reproduced from [60], with permission from American Chemical Society, 2020.

The experimental dynamic surface tensions obtained for Na-HA solutions with concentrations equal to 2.0, 2.8, and 3.5 mg/mL are shown on Figure 10.

Solutions with polymer concentrations equal to 2.0 and 2.8 mg/mL have an induction period, which could be determined by maintaining the surface tension approximately at its the initial level. Then during the formation of a full monolayer (multilayer), a decrease in surface tension is evident, which indicates the achievement of the mesoequilibrium value. During this period (named the mesoequilibrium surface tension regime), molecular reorientation and conformational changes in the absorbed molecules take place, after which the stationary state happens. 

By contrast, solutions with higher polymer concentration (3.5 mg/mL) have significant quantities of molecules, which hinders quick absorption at the air/solution interface. Full surface coverage takes place because of the strong side chain interactions at the interface with the atmosphere. For this reason, the curve does not have an induction period. 

The next study [62] demonstrated a typical plot of the surface tension of high molecular weight hyaluronic acid (Mη = 2100 kDa, *M*_W_ = 5560 kDa) with concentrations from 0.01 μg/mL to 1 mg/mL. Note that in this study, the authors employed two definitions of molecular weight: Mη—molecular weight determined by viscometry (this datum is usually listed in the quality certificate of polymers); and MW—weight average molecular weight determined by light scattering. 

Biopolymer obtained from Genzyme Corporation was dissolved in neutral salt buffers containing deionized water, 0.85% (*w*/*v*) sodium chloride, 0.028% (*w*/*v*) disodium hydrogen phosphate, and 0.004% (*w*/*v*) sodium dihydrogen phosphate monohydrate (pH = 7.2). The experiment showed that no significant differences could be observed between biopolymer solutions with different concentrations of both low molecular weight (Mη = 700–900 kDa, *M*_W_ = 807 kDa) and high molecular weight (Mη = 2100 kDa, *M*_W_ = 5560 kDa) hyaluronic acid solutions. The measured surface tension was equal to 70.00 ± 2.25 mN/m (25 °C) for both types of hyaluronic acid. This value of surface tension is in agreement with the above-mentioned study. 

The authors also used the 1:10 dilution of the ophthalmological specimen named Healon^®^ (10.0 mg/mL, *M*_W_ = 4280 kDa, *M*_n_ = 1300 kDa [63]). In this case, surface tension was equal to 62.7 ± 6.51 mN/m [64]. By contrast, the surface tension of deionized water is equal to 72.17 ± 0.58 mN/m [65], which is favorable to the surface action of hyaluronic acid. 

Johannes Nepp et al. [66] analyzed the viscoelastic properties of artificial tears, including those ones containing sodium hyaluronate with different molecular weights and concentrations: 0.25% (Hyalodrops, Croma, Leobendorf, Austria) with molecular weight 1000 kDa, and 0.4% (Healons, Pharmacia, Stockholm, Sweden) with molecular weight 5000 kDa. The authors measured different properties of artificial tears, and the surface tensions at 18 °C were equal to 73.3 mN/m and 64.9 mN/m, respectively. 

### 2.4. Cohesive and Adhesive Properties

The cohesive properties of a polymer directly depend on its solubility in liquids (organic and non-organic). The cohesive properties of a polymer and compounds based on it are quantitatively related to the cohesive energy. This value is narrowly aligned with the internal pressing, a parameter included in the equation of state of the matter [67]. 

**Figure 11:**
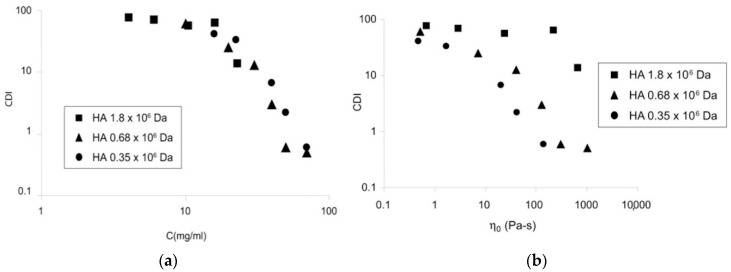
The CDI versus (**a**) the hyaluronic acid concentration and (**b**) zero shear viscosity. Reproduced from [49], with permission from John Wiley and Sons, 2020.

Irrespective of the fact that hyaluronic acid in solution organizes aggregates or implies polymer networks, it is known that its cohesive nature is a unique property of hyaluronic acid in solution [49]. With the provision of favorable semiquantitative methods based on vacuum aspiration [68], it is possible to analyze the cohesive properties of solutions with different molecular weights of hyaluronic acid [49]. The authors used three ranges of biopolymer: 350 kDa, 680 kDa, and 1800 kDa. Note that Modified Dulbecco’s phosphate-buffered solution was used as the polymer solvent. The cohesion-dispersion index (CDI), as a key cohesive parameter, was analyzed by measuring the amount of the sample aspirated by rising vacuum enclosed to the sample. Figure 11a demonstrates the dependence of the CDI on the polymer concentration. Figure 11b illustrates the CDI versus the zero shear viscosity of the polymer solution. 

The CDI decreases with decredased concentration for all solutions, and the data obtained from different molecular weights of hyaluronic acid is very close; therefore, the authors [49] concluded that CDI was independent of the molecular weight of hyaluronic acid. By contrast, CDI decreased with the decrease in the molecular weight of hyaluronic acid. The cohesive nature of biopolymer solutions depends on the molecular weight of the hyaluronic acid. However, the zero shear viscosity is intrinsically unable to characterize the cohesive properties in the hyaluronic acid solution [49].

Summarizing the above, it is obvious that hyaluronic acid with high molecular weight has a higher cohesive level than hyaluronic acid with low molecular weight. This dependence is associated with the structure, as discussed above. Briefly, polymer chains with high molecular weight hyaluronic acid start to entangle each other, resulting in the formation of a taught three-dimensional structure that holds itself as a complete whole (cohesive gel), especially when it is impacted by external action. By contrast, solutions with lower molecular weight hyaluronic acid do not have the same value of chain entwinement. For this reason, such polymers do not demonstrate cohesive properties at a high level [49].

**Figure 12:**
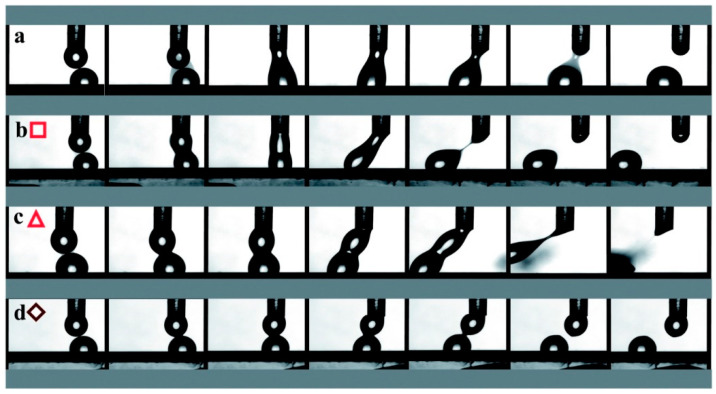
Photos of the hyaluronic acid drops in contact with each other. From left to right: the moment before the touching point, contact point, recession, disassociation. (**a**) 132 kDa, 20.0 mg/mL: (**b**) 1500 kDa 10.0 mg/mL, (**c**) 1500 kDa 20.0 mg/mL, (**d**) 2000 kDa 30 mg/mL. Reproduced from [69], with permission from Royal Society of Chemistry, 2020.

The interfacial behavior of hyaluronic acid upon contact with itself is no less remarkable. To this end, Katherine Vorvolakos et al. [69] used three fractions of hyaluronic acid in contact with itself: 132 kDa (20 mg/mL), 1500 kDa (10 mg/mL and 20 mg/mL), and 2000 kDa (30 mg/mL). Figure 12 demonstrates the behavior of hyaluronic acid drops when contacting each other. Note that the volume of each drop was approximately equal to 1.0 μL and the velocity was equal to 3.0 mm/s. Each image captured 0.033 s. The time between images was selected individually for each sample.

These photos were taken in order to predict dominant characteristics in an interfacial event. Solution A shows rapid droplet coalescence and equally rapid rupture. It has a low viscosity and the dominant characteristic in the interfacial interaction is surface tension. Solution B demonstrates slower coalescence and slower separation, with greater deformation of the droplet and a sluggish recovery to a spherical shape. Here, the surface tension and viscosity of the solution seem equally significant. Solution C has a significantly slow coalescence and significant deformation before uneven separation, spherical curvature is not restored, and drops merge to a lesser extent. Viscosity is the dominant characteristic in this case. Solution D demonstrates the preservation of a spherical shape for almost the entire time of interaction, only slightly deforming the edges of the droplets. 

Yet in view of biomedical applications, the bioadhesive or mucoadhesive properties of hyaluronic acid are more interesting than simple adhesion. The term bioadhesive describes the interactions of two biomaterials with each other or the adhesion between any non-biological agent and a biomaterial [70]. Mucoadhesive, more specifically, shows the sticking ability of the materials with the surface of mucous membrane [71]. Obviously, that hyaluronic acid demonstrates unique mucoadhesive properties [72]; however, they exhibit a positive correlation with the hyaluronic acid and the negative relationship with pH. Thus, the increased molecular weight of the hyaluronic acid and the increase in the acidic content in the solution results in the increase of mucoadhesion [73,74].

### 2.5. Density

As mentioned above, rheological and structural properties of hyaluronic acid are the main subject of interest of the vast majority of scientific papers. In the meantime, the density of HA solutions also attracts attention because of the connection with surface tension and several specific volume characteristics such as partial specific volume and ultrasound velocity. Moreover, density data could help understand the behavior of hyaluronic acid solutions and the structure of the biopolymers.

Thus, in the study [75], the authors measured the density of HA sodium salt (*M*_W_ = 1500 kDa) in aqueous solution at various pH values and in the presence of inorganic salts. In some cases, they also analyzed the effect of temperature. Unfortunately, the main authors’ aim was the establishment of the partial specific volumes at infinite dilution, and the density data are not emphasized.

**Figure 13:**
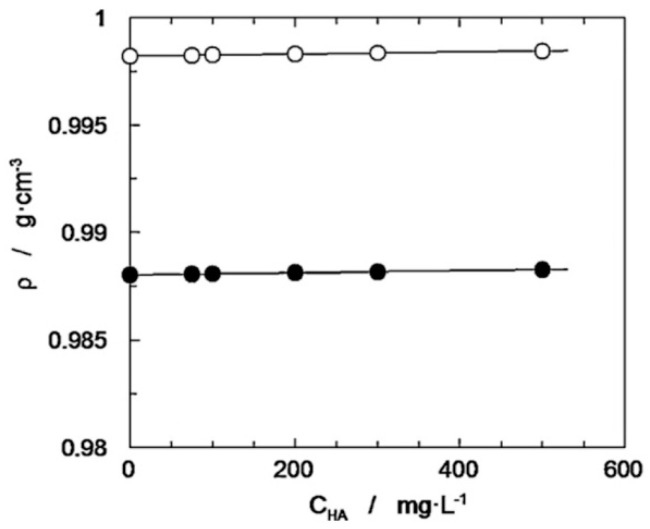
Dependence of the density on hyaluronic acid concentration and temperature: (○) T = 20 °C, (●) T = 50 °C. Adapted from [76], with permission from Elsevier, 2020.

Density data are demonstrated in the next paper [76], but the main goal in that case was to perform viscosity measurements of aqueous and aqueous-alcohol hyaluronic acid solutions. There was only one sample of HA with a determined viscosity-average molecular weight equal to 1430 kDa. The density was measured only for five concentrations, without detailed analysis. Figure 13 shows the influence of biopolymer concentration on solution density at 20 °C and 50 °C.

With reference to Figure 13, the dependence of the density on the temperature is also demonstrated: the influence is much more noticeable than the effect of the biopolymer concentration, and the temperature decrease causes an increase in density, which is obvious.

Additionally, the ultrasound investigations are related to the measurements of density. There have been some studies on this topic [77,78,79], but they have other purposes and the comparable density data of the biopolymers is incomplete. 

Nonetheless, A. Kargerová and M. Pekař [80] reported, perhaps, the first detailed study dedicated to the densitometry of aqueous solutions containing hyaluronan with different molecular weights. To this end, the authors used three ranges of hyaluronan molecular weights: 10–30 kDa, 110–130 kDa, 300–500 kDa and 1500–1750 kDa for density determination. Deionized water and 0.15 M NaCl solutions were used as solvents. The ultrasound velocity was measured separately from the density due to the equipment employed.

**Figure 14:**
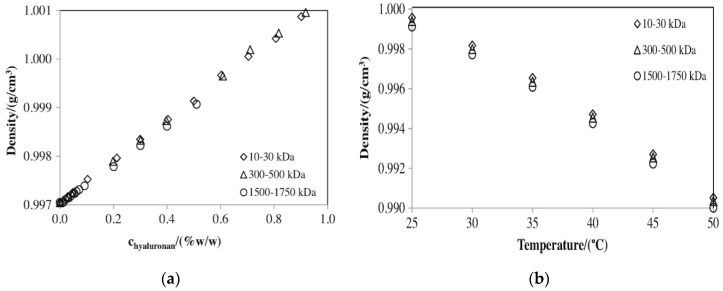
Dependence of density on (**a**) hyaluronic acid concentration and (**b**) temperature. Molecular weights of hyaluronan are as follows: ◊ 10–30 kDa; ∆ 300–500kDa; ○ 1500–1750 kDa. Reproduced from [80], with permission from Elsevier, 2020.

Figure 14a shows that the density has a direct dependence on the hyaluronan concentration at 25 °C. By contrast, as shown in Figure 14b, the density has a negative relationship with temperature (hyaluronan concentration is equal to 0.5% (*w*/*w*)). 

As reflected by Figure 14a and the Supplementary Information of the abovementioned paper, the dependence had a linear character at all tested temperatures. However, there were no significant differences between the densities of solutions with differing molecular weights of hyaluronan. By contrast, the temperature dependence (Figure 14b) was modestly curved and deviated from a straight-line relation. This fact could be explained by the curvilinear character of the temperature dependence of the density of deionized water [81]. The authors also suggested two simple equations to combine the density, concentration, temperature, and molecular weight of hyaluronan. Because of indifference as to the effects of the molecular weight of hyaluronan, the whole data set could be summarized in a single common equation, which would help to calculate the density of hyaluronan solutions with concentrations from 0.0 to 2.0% (*w*/*w*), molecular weights from 10 to 1750 kDa, and temperatures from 25 to 50 °C. 

### 2.6. Ultrasound Velocity

A study of ultrasound velocity under different conditions could be useful for detecting both structural and physical properties, as well as their changes. For example, macroscopic and microscopic structural alterations during crosslinking, glass transition, crystallization, and physical or chemical changes could be analyzed by ultrasound detection without damaging the samples [82].

The sound velocity of polymer solutions is not dependent on the molecular weight of the polymer; however, this affirmation is correct only for frequencies measured in the megahertz diapason. Sound velocity and adiabatic compressibility are not directly dependent on the expansion parameter of the polymer chains in solution, but rather on the interaction of the polymer chains and solvent, the solvation of the polymer segment, and, finally, on the stiffness of the polymer chains [83].

**Figure 15:**
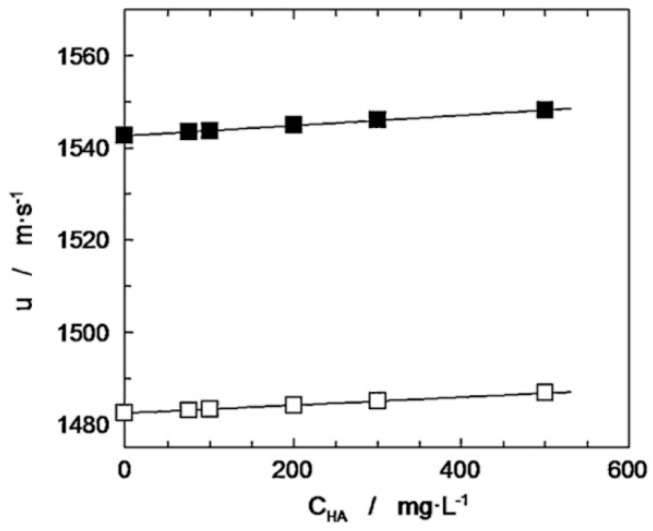
Dependence of ultrasound velocity on hyaluronic acid concentration and temperature: (□) T = 20 °C, (■) T = 50 °C. Adapted from [76], with permission from Elsevier. 2020.

The ultrasound velocity of hyaluronic acid has a lineal dependence on concentration, similar to a variety of other polymer solutions at low concentrations [84]. Figure 15 shows the influence of HA (*M*_W_ = 1430 kDa) concentration on the ultrasound velocity of polymer solutions at 20 °C and 50 °C [76].

The increase in polymer concentration causes a slight increase in ultrasound velocity. This could be related to weak molecular interactions: polymer–solvent and polymer–polymer. The influence of the temperature on the sound velocity is also demonstrated. This parameter has a much greater effect than the concentration; the higher the temperature of the solution is, the greater the ultrasound velocity.

**Figure 16:**
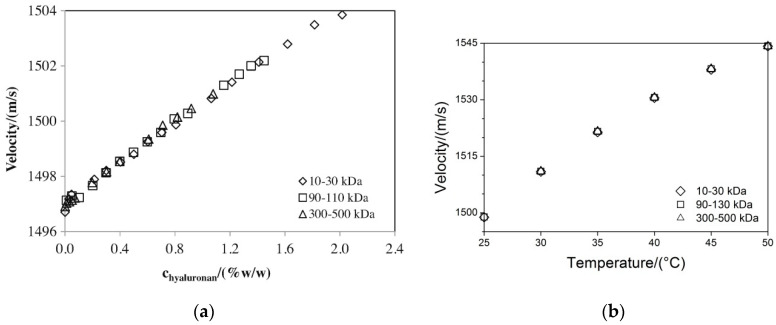
Dependence of ultrasound velocity for (**a**) hyaluronic acid concentration at 25 °C [80] and (**b**) temperature at 0.5% (*w*/*w*) concentration of hyaluronan (based on the Supplementary Materials of [80]). Adapted from [80], with permission from Elsevier, 2020.

The next study [80] confirmed the direct relation between sound velocity and hyaluronan concentration (see Figure 16a). However, the dependence on temperature at a given concentration (see Figure 16b) also has a direct, but slightly curved character that is in agreement with the temperature dependence of sound speed in water [81]. Thus, no significant effect of hyaluronan molecular weight was presented.

The increase of ultrasonic speed with the increase in hyaluronan concentration can be explained by solubilized polymeric chains and the hydration pellicle encapsulated around them. The greater the stiffness of the sample, the greater the velocity of ultrasound transmission. Bulk water is known to have higher compressibility than hydration water [79]. For this reason, the ultrasound velocity in systems with hydrated molecules is greater. As mentioned above, the effect of concentration of hyaluronan is lower than the effect of temperature [76], which could be related with the dependency of the specific compressibility of water on temperature. The slight impact of hyaluronan molecular weight on ultrasound velocity and density is aligned with the major role of hyaluronan elementary disaccharide unit in the uncovering of polymer solution properties. The molar amount at a fixed concentration of hyaluronan is divorced from the molecular weight of the biopolymer [80].

### 2.7. Osmolality and Colloid Osmotic Pressure

It is known that the osmolality of any solution is the quantity of unentangled particles per 1 kg water in Mol and expressed in Osm/kg H_2_O. In comparison, osmolarity demonstrates the number of unrelated particles per liter of solution in Mol (Osm/L). Within the meaning of the physiology of biological fluids, the divide between osmolality and osmolarity is insignificant due to the dilute aqueous nature of body fluids; however, in healthcare and biochemistry, the term osmolality is usually used. The solution is named hyperosmotic if the osmolality is higher than that of the compared biological fluid, and hypoosmotic if the osmolality is less than that of the mentioned solution. The isosmotic solutions have an osmolality equal to that of body fluid [85].

Meanwhile, osmolality is very important, especially for developing medical applications and artificial human liquids, because of the limited range of osmolality of body fluids and the terminated tolerance of human tissues to affecting endogenous or exogenous liquids. For example, osmolarity and osmolality of natural tears are approximately equal to 304 mOsm/L [86,87] and 304 mOsm/kg [88], respectively. Note that tear hyperosmolality could cause a negative effect on the ocular surface, consisting of the initiation of inflammation and increasing of lacrimal film instability [89]. However, there are some artificial tears which have special hypotonic formulation [90]. It is recognized that hypotonic aqueous solutions could be useful for dry eye syndrome when the tears are characterized as hyperosmotic [91,92].

The cornea and aqueous fluid have osmolality equal to 305 mOsm/kg and 300 mOsm/kg, respectively [93]. The higher the osmolality of an artificial medical substance in connection with the tissue fluid (300 mOsm/kg), the more extensive its capability for the dehydration of adjoining tissues [94]. Note that the human cornea allows standing liquids with osmolality from 200 to 400 mOsm/kg, but endotheliocyte could be deteriorated by fluids whose osmolality is low [95]. Thus, exposing a healthy cornea to hypo-osmotic fluid (240 mOsm/kg) causes swelling of the endothelial cells due to the osmotic gradient; however, without any long-term damages [96]. By contrast, if the cornea is short on endothelial cells or has a functional anomaly, contact with hypo-osmotic fluid could cause major changes to the corneal edema [97]. For these reasons, osmolality is one of the key parameters of artificial human fluids and biomedical substances. This parameter should be steady within the range from 250 to 400 mOsm/kg [94].

Hyaluronic acid, being a popular component of artificial tears and medical substances, as much as the other glycosaminoglycans, could increase the osmolality of solutions. The greater the concentration or the higher molecular weight of HA, the greater the osmolality of the solution [98]. With this in mind, the manufacturers have to elaborate special formulae of their products; however, the osmolality of such products is not easy to analyze and compare, due to the variety of different hyaluronic acid polydispersity indexes, different buffering solutions, addition polymers, preservatives, surfactants, organic osmolytes, etc. [99]. Nonetheless, the brief summary based on [93,94] and provided in Table 4 generally confirms the direct dependence of osmolality on HA concentration and molecular weight.

Interestingly, in a recent study [100], two different types of ophthalmic solution (HA: 0.30% aqueous solution of hyaluronic acid; and TH-HA: the blend of trehalose 3.00%, hyaluronic acid 0.15%, and carbomer 0.25%) were compared. The authors noted that the continuation of the solutions when maintained on the ocular surface was different, and the HA solution had a greater long-term effect than the TH-HA solution. Moreover, this effect was associated with longer feelings of comfort in the patient.

One more parameter is essential to biological fluids and is required for each artificial substance and biomedical product. This refers to osmotic pressure, and, more specifically, to colloid osmotic pressure. Osmotic pressure mostly depends upon the concentration of inorganic salts; on the other hand, the colloid osmotic pressure is sensitive to concentration, type of polymer, molecular weight, and the degree of cooperation between the polymer molecules. In solutions with mucopolysaccharides, with increasing polymer concentration, the osmotic pressure of the colloid rapidly increases, as well as osmolality.

**Figure 17:**
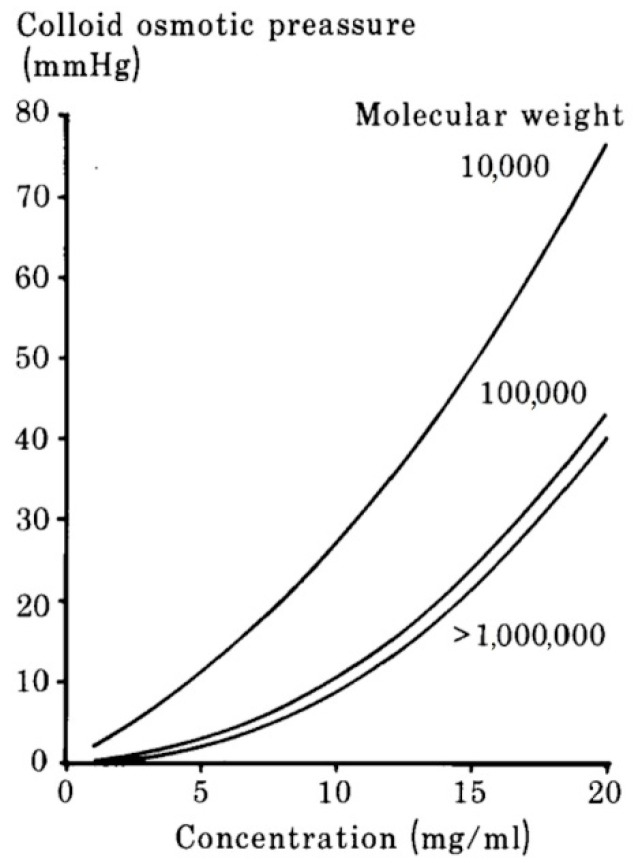
Dependence of the colloid osmotic pressure of hyaluronate solutions on concentration and molecular weight. Adapted from [50], with permission from Taylor & Francis, 2020.

The colloid osmotic pressure in hyaluronate solutions is demonstrated in Figure 17 as a function of biopolymer concentration. Note that the hyaluronate colloid osmotic pressure is not dependent on molecular weights greater than 100 kDa, or the very rapid increase of colloid osmotic pressure with biopolymer concentration [36]. Solutions with low molecular weight hyaluronate achieve a colloid osmotic pressure of 30 mmHg (physiological level of plasma) at concentrations equal to 10 mg/mL. By contrast, solutions consisting of high molecular weight hyaluronate possess the same osmotic pressure at concentrations of 15 mg/mL [94].

It is critical to correctly choose the colloid osmotic pressure of biomedical products used. When the colloid osmotic pressure of hyaluronic acid solution is greater than that of the surrounding tissues (or body fluids), water infiltrates into the biopolymer network until the level of the colloid osmotic pressure of the resulting polymer solution is equal to the osmotic pressure of the surroundings. Thus, the concentration of the inserted solution with low molecular weight, HA, momentarily decreases from 40 mg/mL to 10 mg/mL, and the solution viscosity falls to the lowest level. For this reason, it is preferable to use highly viscous hyaluronic acid solutions based on high molecular weight hyaluronic acid at concentrations equal to 10 mg/mL instead of solutions with low molecular weight, HA, and at higher concentrations [50].

### 2.8. Hydraulic Conductivity and Fluid Absorption Rate

Tissue hydraulic conductivity in general and peritoneal tissue hydraulic conductivity in particular play a key role in peritoneal fluid (from peritoneal cavity to blood and vice versa) and transport of solutes. Hyaluronan is a major asset in tissue hydraulic conductivity and demonstrates the high barrier properties with respect to water flow [101]. Hyaluronan could act as a “tissue screen” and protect living tissues from the significant alterations in aqueous content [20], preventing the efflux from the peritoneum. Note that the effect of hyaluronan depends not only on the molecular weight of hyaluronan, but also on the biopolymer concentration [102].

The first study of sedimentation velocity which could be used for further equitation of hydraulic conductivity (as a parameter binding the pressure gradient and fluid flow) of hyaluronic acid solutions was performed in 1960 by Torvard C. Laurent et al. [103]. It was revealed that the concentration dependence of sedimentation velocity is higher at low polymer concentrations. At high concentrations, the velocity of sedimentation does not depend on molecular weight, which is attributable to the absence of single molecule sedimenting; however, the three-dimensional network is formed.

**Figure 18:**
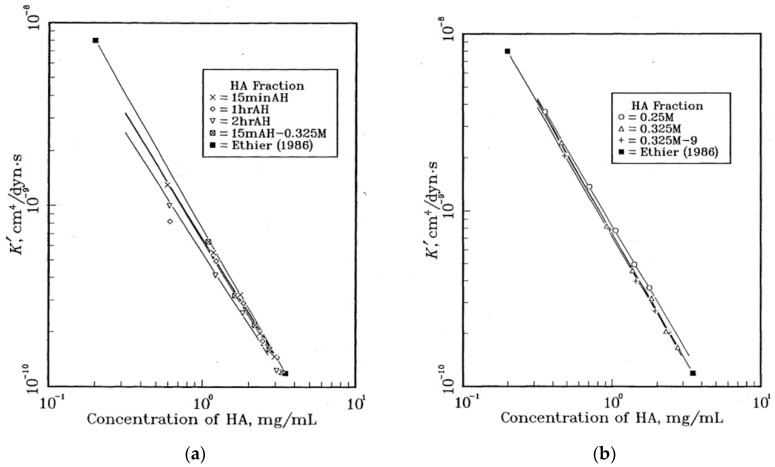
Dependence of hydraulic flow conductivity of hyaluronic acid solutions on biopolymer concentration, where HA has a variety of molecular weights: (**a**) 15minAH — 165 kDa, 1hrAH – 81.9 kDa, 2hrAH – 45.4 kDa, 15minAH—0.325M – 196 kDa, Ethier (1986) [106]; (**b**) 0.25M – 699 kDa, 0.325M – 844 kDa, 0.325M—9 – 1110 kDa, Ethier (1986) [106] (reproduced from [104], with permission from University of British Columbia, 2020).

The next studies [104,105] continued the investigation of flow conductivity of hyaluronic acid solutions. The authors used seven samples of hyaluronic acid with different molecular weights and compared their results [106]. Figure 18a,b demonstrates the dependence of hydraulic flow conductivity on hyaluronic acid concentration.

The flow conductivity decreases with polymer concentration, which is related to the enlargement of the entanglement between the hyaluronic acid molecular chains [50,54]. However, the differences between hydraulic conductivity are smoothed over at high concentrations because of the similitude of polymer chain entwinement and the formation of the three-dimensional polymer network.

When comparing the low molecular weight samples (Figure 18a) and the high molecular weight samples (Figure 18b), it becomes apparent that at low concentrations, the first range has a lower level of hydraulic conductivity than the second range. This could be explained by the compactability of the polymer molecules in high molecular hyaluronic acid solutions, resulting in a decrease in the flow resistance to the solvent flow. Moreover, in the case of the same low concentrations, there are fewer high molecular weight molecules than low molecular weight ones, and the entanglement degree of the latter is greater than the former. For this reason, the resistance to the solvent flow is higher at higher degrees of entanglement [104].

**Figure 19:**
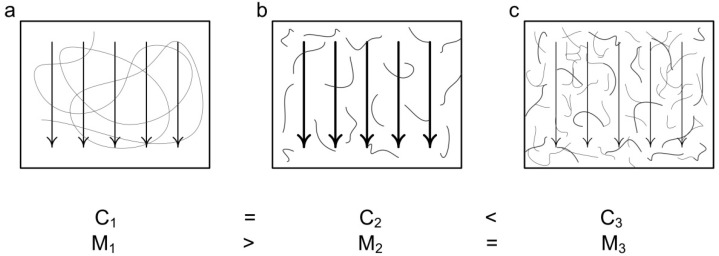
Stylized representation of the effect of various concentrations and molecular weight of hyaluronic acid on the floe conductivity. (**a**) C_1_ = C_2_, M_1_ > M_2_, (**b**) C_2_ = C_1_, M_2_ < M_1_, (**c**) C_3_ > C_2_, M_3_ = M_2_. Here C_1_, C_2_, and C_3_—hyaluronic acid concentrations, M_1_, M_2_, M_3_—molecular weight of hyaluronic acids. (Redrawn from [102]).

The next study [102] is not directly devoted to hydraulic conductivity, but describes the peritoneal fluid absorption and fluid absorption rate, which directly depend on flow conductivity. Thus, the higher the tissue hydraulic conductivity is, the higher the fluid absorption to peritoneal tissues. Furthermore, the lower the viscosity of fluid in the cavitas peritonealis, the higher the rate of the fluid absorption [49,107]. Wang T. et al. [102] confirmed that the high molecular weight hyaluronic acid could decrease the flow conductivity more significantly, but meanwhile it could decrease the ultrafiltration of the transcapillary. In Figure 19, the effect of the molecular weight of hyaluronic acid on the flow conductivity is demonstrated.

The difference between the behaviors of hyaluronic acid with various molecular weights depends on the inequality of the structure of the hyaluronic acid molecular chains [51]. On the one hand, hyaluronic acid in solution could organize a three-dimensional cellular structure with enormous dimensions at a concentration of less than 1 μg/mL [34]. On the other hand, the HA molecules, being interwined coils, could entangle at concentrations 1 mg/mL or even less [19,20]. It is known that the process of entanglement significantly depends on the molecular weight of the biopolymer, and has less dependency on the concentration of the lower molecular weight solutions [51]. An extensive random polymer molecule involves large volume and prevents a lot of flow directions (Figure 19a). By contrast, hyaluronic acid macromolecules with low molecular weight are affected by the flow with higher degree (Figure 19b). With the increase in concentration (Figure 19c), the internal friction increases [50]. The intertangled network has a tough structure, and for this reason has a better fluid flow resistance through the layer [50].

### 2.9. Section Summary

A brief summary overview of the scientific sources is provided in Table 5.

## 3. Degradable Properties

The presence of hyaluronic acid in many tissues and fluids determines its widespread use in medicine and cosmetology. The biological activity of HA depends on its molecular weight [108]. It has been shown that high molecular weight HA has anti-inflammatory properties, and its rheological characteristics determine its use as a synovial fluid prosthesis in the treatment of various joint diseases, in cosmetology, and in aesthetic medicine as dermal fillers and in ophthalmology as artificial tears [6,109]. Degradation of HA leads to a decrease in the molecular weight and, consequently, to a decrease in viscosity, which is detrimental to the use of HA [40,53].

### 3.1. Degradation by Ultrasound

**Figure 20:**
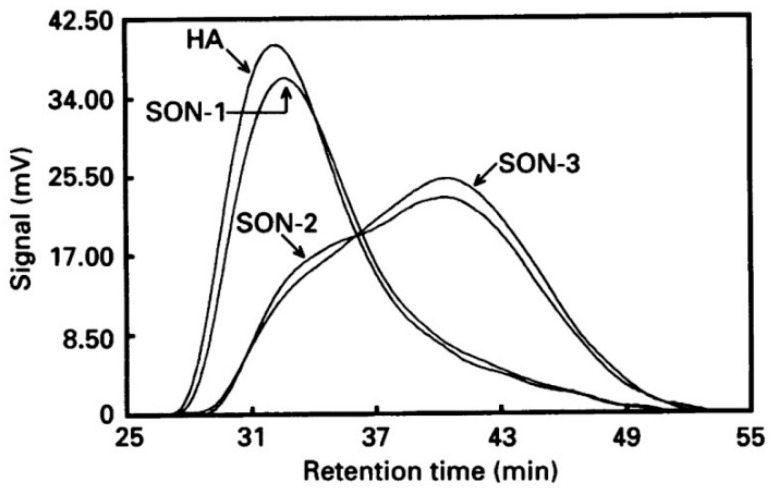
Chromatograms of HA exposed to ultrasound for 0 (HA), 15 (SON-1), 45 (SON-2), 60 (SON-3) minutes. Reproduced from [110], with permission from Portland Press, Ltd., 2020.

Hyaluronic acid undergoes degradation under the influence of ultrasound [110,111,112]. This happens as a result of a cleavage of the glycosidic bonds between GlcA and GlcNAc units by the free radicals OH and H, which can be generated by the action of ultrasonic waves in water and the collapse of cavitation bubbles, which causes the breakage of the macromolecule backbone in the solutions [111]. Interestingly, sonication leads to the degradation of HA in a non-random fashion (Figure 20). It is assumed that high molecular weight HA degrades more slowly than low molecular weight HA due to the bimodal shape of the chromatograms obtained after 45 and 60 min of sonication [110]. Unfortunately, this study does not provide detailed information for either the MW of HA or the power of sonication used. Kubo et al. concluded that the action of ultrasound did not lead to the degradation of HA to monomers or oligosaccharides (Table 6). Initially, the molecular weight of HA samples obtained from different sources was 400 kDa, 1000 kDa, and 1200 kDa; after 10 h of exposure to ultrasound, the molecular weight dropped to 11 kDa, 3 kDa, and 60 kDa, respectively [112]. Sonication was performed at 20 kHz, 7.5 W at 0 °C.

### 3.2. Change of pH of a Solution

Exposure to alkali and acid also leads to the degradation of hyaluronic acid [43,113,114]. This method leads to the complete hydrolysis of HA to oligosaccharide-hyalobiuronic acid [113]. With the presence of acid, hydrolysis randomly occurs on glucuronic acid, and under the action of alkali, it randomly occurs on acetylglycosamine [114]. It is hard to assume that there is any cohesion between the rate of degradation and molecular weight of HA; however, it is suggested that the pH value, as along with the concentration of HA, may affect the rate of hydrolysis [42].

### 3.3. Thermal Degradation

Presumably, the mechanism of thermal degradation of HA is a random chain scission that occurs in the HA chain [115,116,117,118]. Ref. [115] studied the degradation of samples of HA of two MW-1670 kDa and 1800 kDa, both in aqueous solution and in the form of a powder. After being subjected to a temperature of 37 °C for 4 h, the loss of MW in the solution was 8.6% and 3%, respectively, but after 12 h, the percentage difference in loss of MW was almost equal. With increasing temperature, the decrease in molecular weight was more rapid for both the sample in solution and the powder. During the first three hours of heating at a temperature of 90 °C (powder and solution) and 120 °C (powder), the decrease in molecular weight was much more instantaneous than with a longer exposure to lower temperature. In general, it was concluded that degradation of HA with a lower MW occurs more quickly than with a higher MW at a moderate temperature (3 h, 60 °C: 11.4% loss in powder and 7.7% in solution for 1670 kDa and loss 7.3% in powder and 3.4% in solution for 1800 kDa).

### 3.4. Oxidative Degradation

It is known that HA degrades when exposed to reactive oxygen species. The impact of various oxidizing agents such as ozone, UV light, hydrogen peroxide and others on HA was studied [119,120,121,122,123,124,125]. Unfortunately, there is no information available about the dependence of the rate of oxidative degradation of HA on its molecular weight as only one sample of HA was studied in most articles.

### 3.5. Long-Term Degradation Caused by Storage Time

**Figure 21:**
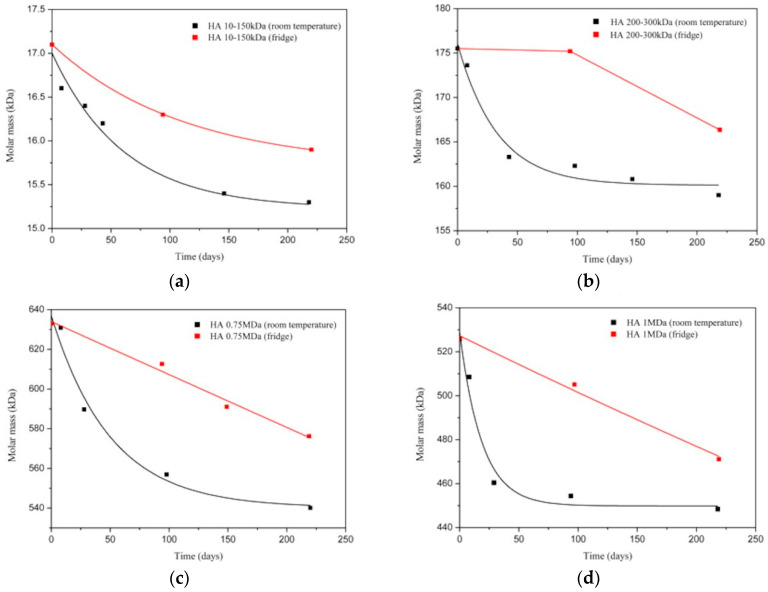
Degradation of HA samples with different MW: (**a**) 17 kDa, (**b**) 267 kDa, (**c**) 752 kDa, (**d**) 1000 kDa. Reproduced from [126], with permission from Elsevier, 2020.

Hyaluronic acid undergoes degradation under normal conditions. To minimize molecular weight loss during long-term storage, HA can be put in the refrigerator. In [126], the molecular weight loss of four HA powder samples was studied (Figure 21):

- HA1 MDa: MW 1000 kDa;
- HA 0.75 MDa: MW 752 kDa;
- HA 200–300 kDa: MW 267 kDa;
- HA 10–150 kDa: MW 17 kDa.

It was noted that the samples were stored under different conditions before the experiment. The sample with the greatest MW had been stored in the refrigerator for 6 years before the research was conducted, while the last sample had been purchased several weeks before the study, leading to loss of MW of the first sample.

It is expected that degradation will be faster at room temperature for all samples. This conclusion indicates that storage conditions have a greater effect on degradation than the initial molecular weight of the sample. In general, the authors reported weight loss of about 9–15% at room temperature and 5%–10% in the refrigerator.

Another work [127] also investigated the degradation of HA at room temperature and in the refrigerator, but in an aqueous solution. The following samples were taken: 14.3 kDa, 267.2 kDa and 1160.6 kDa, measured for fresh solutions before degradation. No ingredients were added to the solution in order to protect against exposure to microorganisms. After two months of storage of the solutions at room temperature or in a refrigerator, the following results were obtained: average molecular weight decreased by 90% for 14.3 kDa, 95% for 267.2 kDa and 71% for 1160.6 kDa hyaluronan (room temperature) or 5.6% for 14.3 kDa, 6.2% for 267.2 kDa and 7.7% for 1160.6 kDa hyaluronan (refrigerator temperature).

An interesting dependence is visible here—the degradation of low molecular weight HA occurs faster in solution when stored in air, but slower when stored in the refrigerator.

### 3.6. Enzymatic Degradation

HA has a high turnover rate at the cellular and tissue levels, mainly due to enzymatic hydrolysis of hyaluronidases (HYALs), which include HYAL1, HYAL2, HYAL3, HYAL4, HYALP1 and PH-20 [128]. Advantageously, HYAL1 and HYAL2 are considered major HA-degrading enzymes in somatic tissue.

HMW HA is bound to its receptor CD44 on the cell surface, where HYAL2 is located, and it is capable of cleaving HMW HA chains of up to 20 kDa, which corresponds to a chain of approximately 50 disaccharide residues. These chains are then internalized and transformed to lysosomes, where HYAL1 further cleaves HA to tetrasaccharides [109,129].

The study of the biodegradation of hydrogels of various compositions based on HA is currently receiving attention. Hydrogels of HA can be applied in different fields, including tissue engineering, drug delivery, wound dressings and regenerative medicine due to its biodegradability, biocompatibility and versatility [130]. To obtain hydrogel from HA, the latter might be crosslinked by chemical modification. In addition to creating a three-dimensional structure, chemical modification makes it possible to achieve better physicochemical characteristics in hydrogels, thereby increasing their resistance to biodegradation [131].

**Figure 22:**
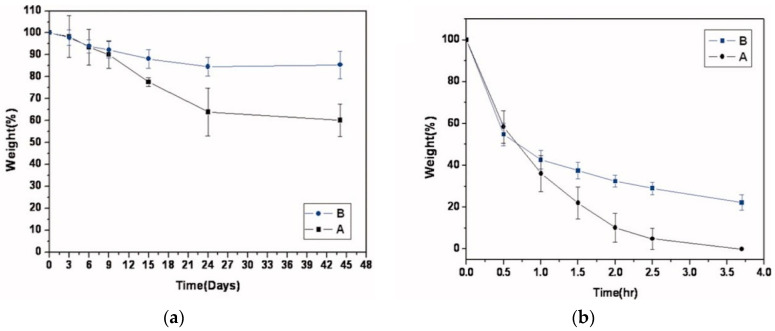
In vitro degradation rate of HA hydrogels: (**a**) degradation by hydrolysis, and (**b**) degradation by hyaluronidase. A: 10 kDa, B: 50 kDa. Reproduced from [132], with permission from John Wiley and Sons, 2020.

In [132], two samples of HA with MW 10 and 50 kDa were used to prepare hydrogels through acrylation of hyaluronic acid. The authors suggested that degradation of those gels occurred in two steps: hydrolysis, due to the ester linkage in the crosslinking bonds; and biodegradation of hyaluronic acid by hyaluronidases. Both hydrogels were exposed to PBS (phosphate buffer solution) and to 100 U/mL hyaluronidase (dissolved in PBS also) (Figure 22).

As can be seen from the figure, both hydrogels retained more than half of their weight, even after 45 days in PBS; however, gels with higher molecular weight were less susceptible to degradation. In the presence of hyaluronidase, both gels underwent almost complete degradation after 3.7 h, yet the gel with higher MW retained more of its weight.

Another study [133] prepared five samples of HA-based hydrogels using different ratios of high and low molecular weight HA (2000 kDa and 200 kDa). 1,4-Butanediol diglycidyl ether (BDDE) was used as a crosslinking agent. The compositions of all hydrogels are listed in Table 7.

**Figure 23:**
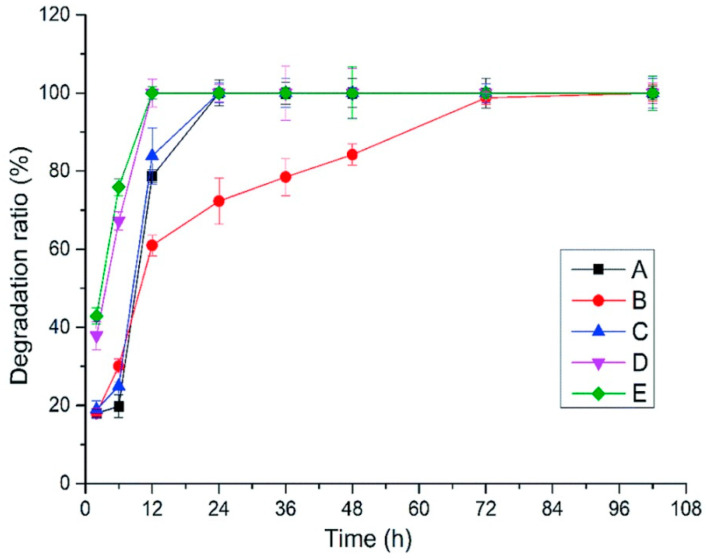
Degradation of crosslinked hydrogels A–E prepared at different mixing ratio in vitro. Reproduced from [133], with permission from The Royal Society of Chemistry (RSC), 2020

To study enzyme hydrolysis stability, all gels were swollen in PBS for 12 h, then mixed with 10 mL hyaluronidase solution (100 U mL^−1^, dissolved in PBS), and then incubated at 42 °C (Figure 23).

Hydrogels D and E were completely degraded after 12 h, C and A were degraded after 24 h, and hydrogel B was degraded after 72 h. It is clear that increase in LMW HA increases degradation rate, and degradation of hydrogel containing LMW HA alone was the fastest. Interestingly, degradation of hydrogel composed of HMW HA alone was not the slowest. System B, with a mass ratio of HMW HA to LMW HA of 4:1, had the best anti-enzymatic hydrolysis performance. The authors suggest that during the preparation of the hydrogels, the intramolecular hydrogen bonds of both samples of HA were disturbed, thus leading to entanglement of chains and the creation of cooperative complexes. This phenomenon was previously explained by A. Stellavato et al. [134].

**Figure 24:**
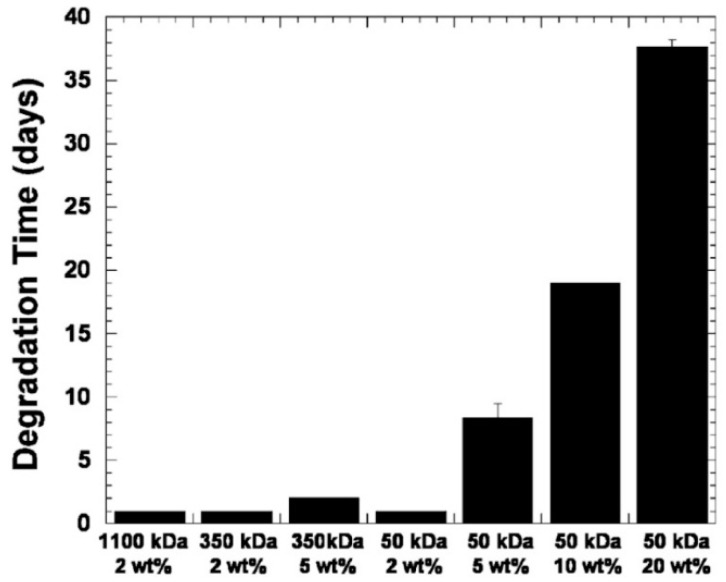
Time of complete degradation of MeHA-based hydrogels in a solution of 100 U/mL hyaluronidase in PBS. Reproduced from [135], with permission from American Chemical Society, 2020.

Interesting results were obtained by J. Burdick et al. [135]. In this work, hydrogels were synthesized from hyaluronic acid (MW = 50 kDa, 350 kDa, 1100 kDa), which was modified with methacrylic anhydride and then photopolymerized. Macromer concentrations varied from 2 to 20 wt.%. Hyaluronidase concentration was 100 U/mL, dissolved in PBS (Figure 24).

Time of complete degradation of hydrogel composed of 350 kDa HA, 5 wt.% was less than for hydrogel composed of 50 kDa HA, 5 wt.%. The authors note the correlation between the degradation time and the swelling ratio. This is true for hydrogels obtained from HA samples of the same molecular weight with different concentrations, although the swelling ratio of hydrogels with different molecular weights but the same concentration differed insignificantly.

**Figure 25:**
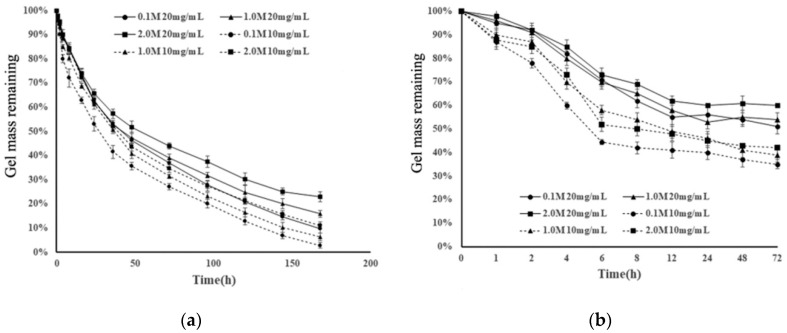
In vitro degradation of HA-SH-Ac-HA hydrogels by hyaluronidase (**a**) and by DTT (**b**). Reproduced from [136], with permission from Royal Society of Chemistry, 2020.

Different results were obtained in another study [136]. Hydrogels were composed of thiolated HA (HA-SH) and acrylated HA (HA-Ac). Degradation was performed in solution of hyaluronidase (50 μg mL^−1^) in PBS and in PBS solution containing dithiothreitol (0.1 mM) (Figure 25).

In this paper, the cohesion between the swelling ratio and degradation was also observed. With the increase of MW, single HA molecules form more hydrogen bonds, thus leading to greater entanglement of HA chains, and with addition of cross-linking points, this results in a decrease in the swelling ratio. Degradation rate increases with the decrease of MW and concentration in both cases. In the first case, hyaluronidase cleaves glycosidic bonds in HA chain; however, in the presence of DTT, only disulfide bonds underwent degradation in the first 6–8 h and further degradation was almost imperceptible.

### 3.7. Section Summary

A brief summary overview of the scientific sources is provided in Table 8.

## 4. Conclusions and Perspectives

Hyaluronic acid, as a hydrophilic biopolymer with a unique set of structural, physical, physicochemical, and biodegradable properties, attracts a great deal of attention. This review focuses on the dependency of the key properties on molecular weight of hyaluronic acid. Firstly, the hyaluronic acid structure and coil overlap were discussed. However, despite comprehensive studies, this field requires more detailed analysis, for example, with respect to structural dimensions such as the diameter of the coil, etc.

Secondly, viscosity, surface tension, and density, as the key parameters, were investigated in detail. Further analysis is viable for aqueous-organic solutions of hyaluronic acid or for aqueous HA solutions with additional polymers, which are applied for electrospinning to obtain nano- and microfibers.

Thirdly, knowledge of the cohesion and adhesion properties of hyaluronic acid is necessary for the development of biomedical applications, especially for surgery, ambustial therapy, wound healing, and cell growing. Such parameters were extensively analyzed, but it is interesting to evaluate the influence of the biologically active agents used in the abovementioned applications on the cohesion and adhesion properties of the compound formed.

The next parameter, ultrasound velocity, is not important in itself. Moreover, it has been discovered that this parameter is not dependent on the molecular weight of the polymer. Nonetheless, measuring the ultrasound velocity is useful for the determination of the structural and physical properties of the polymer and their alterations.

Osmolality and colloid osmotic pressure are very important parameters of body liquids. Moreover, during the development of any kind of artificial fluids (tears, synovial fluid, etc.), it is critical to choose an osmolality that is approximately equal to the natural one. Osmolality was analyzed using eye drops, while the colloid osmotic pressure was investigated based on three fractions of hyaluronic acid. Future studies with a wider range of molecular weights could expand the fundamental scientific data in this field.

One more key parameter is hydraulic conductivity, which is as important for peritoneal fluid as it is for transport solutes. Furthermore, this parameter must be considered for the development of medical applications. Unfortunately, there are only a small number of studies in this field, and this area requires additional examination.

Study of the degradation processes of hyaluronic acid under the influence of oxidants and enzymes is necessary for assessing the half-life of drugs based on hyaluronic acid.

It was shown that HA does not degrade to oligosaccharides under the influence of ultrasound; this is in contradistinction to the action of pH and oxidants, which lead to complete hydrolysis of HA.

Moreover, long-term degradation and thermal degradation were discussed. The dependence of these parameters on the molecular weight of the HA makes it possible to choose the optimal period and temperature for storing the sample in order to avoid loss of molecular weight.

Study of the time and degree of enzymatic hydrolysis is necessary for assessing the duration of drug efficacy. It has been shown that hydrogels consisting of HA with a higher molecular weight are less susceptible to enzymatic hydrolysis, although the molecular weight of the sample is not the only factor to affect the degree of decomposition.

However, scientific studies call for further investigations for a better understanding of the relation between the degradable properties and the molecular weight of hyaluronic acid. Such investigations may create a background for development of topical and complicated drug delivery systems, scaffolds and wound dressings, which take biomedicine and bioengineering to a new level.

## Figures and Tables

**Table 1:** Limiting viscosity number and hydrodynamic size of hyaluronic acid with various molecular weights [40].

M, kDa	L, nm	[η], cm^3^/g	V_s_, cm^3^/g	<r^2^>^1/2^, nm	C for Coil Overlap, μg/cm^3^
100	250	290	120	52	8600
500	1250	1100	420	140	2400
1000	2500	1800	730	210	1400
3000	7500	4400	1800	400	570
6000	15,000	7700	3100	600	320

**Table 2:** Viscosity (mPa∙s) of hyaluronic acid solutions with different concentrations and molecular weights. Adapted from [50], with permission from Taylor & Francis, 2020.

Hyaluronic Acid Concentration (mg/mL)	Molecular Weight (kDa)			
	1000	2000	3000	4000
10.0	1500	18,000	89,000	220,000
20.0	18,000	22,000	940,000	2,600,000

**Table 3:** Rheological properties of hyaluronic acid solutions [52].

MW (kDa)	Zero Shear Viscosity (Pa∙s)	0.5 Hz		2.5 Hz		Crossover Frequency (Hz)
		G′ (Pa)	G″ (Pa)	G′ (Pa)	G″ (Pa)	
77	0.013 ± 3 × 10^−3^	-	-	-	-	-
		640	1.67 ± 0.05	5.4 ± 0.3	22.2 ± 0.2	-
1060	11.6 ± 0.4	13.5 ± 1.5	29.0 ± 2.5	55.8 ± 5.6	67.5 ± 5.3	-
2010	107.0 ± 1.7	101.0 ± 3.5	92.3 ± 4.0	220.0 ± 9.5	125.0 ± 6.3	0.4

**Table 4:** Examples of lubricant eye drops and their characteristics.

Eye Drop	Manufacturer	C (HA), %	MW (HA), kDa	Osmolality, Osm/kg
AMO Allervisc	Allergan	1.0	5100	299
AMO Allervisc Plus	Allergan	1.4	5100	307
AMO Vitrax	Allergan	3.0	500	284
Healon	Pharmacia & Upjohn	1.0	4000	295
Healon GV	Pharmacia & Upjohn	1.4	5000	312
Healon5	Pharmacia & Upjohn	2.3	4000	322
Microvisc	Bohus BioTech	1.0	5000	313
Microvisc Plus	Bohus BioTech	1.4	7900	341
Dispasan	Ciba Vision Ophthalmics	1.0	2000	311
Dispasan Plus	Ciba Vision Ophthalmics	1.5	3000	314
Visko	Domilens	1.0	2000	296
Visko Plus	Domilens	1.4	3000	319

**Table 5:** Summary of structural, physical, and physico-chemical properties of hyaluronic acid with various molecular weights.

Property	HA MW, kDa	Authors	Reference
Hydrodynamic size	100, 500, 1000, 3000, 6000	Cowman et al.	[40]
Rheology	350, 680, 1800	Falcone et al.	[49]
	1000, 2000, 3000, 4000	Bothner et al.	[50]
	125, 241, 390, 598, 800, 961, 1270, 1430, 1620, 1770, 2040, 2150	Yanaki and Yamaguchi	[54]
Surface tension	100, 500, 4000	Knepper et al.	[59]
	1630	Ribeiro et al.	[60]
	1500	Krause et al.	[61]
	807, 4280, 5560	Silver et al.	[62,64]
	1000, 5000	Nepp et al.	[66]
Cohesion/adhesion	350, 680, 1800	Falcone et al.	[49]
	132, 1500, 2000	Vorvolakos et al.	[69]
Density	1500	Gómez-Alejandre et al.	[75]
	1430	García-Abuín et al.	[76]
	10–30, 110–130, 300–500, 1500–1750	Kargerová and Pekař	[80]
Ultrasound velocity	1430	García-Abuín et al.	[76]
	10–30, 110–130, 300–500, 1500–1750	Kargerová and Pekař	[80]
Osmolality and colloid osmotic pressure	1000, 2000, 3000, 4000	Bothner et al.	[50]
	From 500 to 7900 (eye drops)	Dick et al.	[93]
		Dick	[94]
Hydraulic conductivity and fluid absorption rate	85, 280, 500, 4000	Wang et al.	[102]
	45.4, 81.9, 165, 196, 699, 844, 1110	Lam	[104]
		Lam and Bert	[105]

**Table 6:** Degradation of HA derived from various sources by sonication. Reproduced from [112], with permission from Springer Nature, 2020.

Source of HA	Molecular Weight, kDa	
	Before	After
Human umbilical cord	400	11
Rooster comb	1000	3
Streptococcus zooepidemicus	1200	60

**Table 7:** Component list and quantities for five hydrogel samples [133].

	A	B	C	D	E
HMW-Ha (g)	0.5	0.4	0.375	0.333	0
LMW-Ha (g)	0	0.1	0.125	0.167	0.5
NaOH (mL)	4.95	4.95	4.95	4.95	4.95
BDDE (μL)	50	50	50	50	50

**Table 8:** Summary of degradable properties of hyaluronic acid with different molecular weight.

Type of Degradation	HA MW, kDa	Author	Reference
Ultrasound	400, 1000, 1200	Kubo et al.	[112]
Temperature	1670, 1800	Mondek et al.	[115]
Long-term (caused by storage time)	17, 267, 752, 1000	Simulescu et al.	[126]
	14.3, 267.2, 1160.6	Simulescu et al.	[127]
Enzymatic	10, 50	Kim et al.	[132]
	200, 2000	Xue et al.	[133]
	50, 350, 1100	Burdick et al.	[135]
	100, 1000, 2000	Cao et al.	[136]
